# All-optical AZO-based modulator topped with Si metasurfaces

**DOI:** 10.1038/s41598-022-25991-9

**Published:** 2022-12-13

**Authors:** Sareh Vatani, Behdad Barahimi, Mohammad Kazem Moravvej-Farshi

**Affiliations:** Nano Plasmo-Photonic Research Group, Faculty of Electrical and Computer Engineering, Tarbiat Modares University, Tehran, 1411713116 Iran

**Keywords:** Metamaterials, Electrical and electronic engineering, Photonic devices

## Abstract

All-optical communication systems are under continuous development to address different core elements of inconvenience. Here, we numerically investigate an all-optical modulator, realizing a highly efficient modulation depth of 22 dB and a low insertion loss of 0.32 dB. The tunable optical element of the proposed modulator is a layer of Al-doped Zinc Oxide (AZO), also known as an epsilon-near-zero transparent conductive oxide. Sandwiching the AZO layer between a carefully designed distributed Bragg reflector and a dielectric metasurface—i.e., composed of a two-dimensional periodic array of cubic Si—provides a guided-mode resonance at the OFF state of the modulator, preventing the incident signal reflection at *λ* = 1310 nm. We demonstrate the required pump fluence for switching between the ON/OFF states of the designed modulator is about a few milli-Joules per cm^2^. The unique properties of the AZO layer, along with the engineered dielectric metasurface above it, change the reflection from 1 to 93%, helping design better experimental configurations for the next-generation all-optical communication systems.

## Introduction

Controlling the light flow using all-optical systems is commonly achieved through nonlinear light-matter interactions. All-optical control as a continuing goal in optics and photonics bypasses all inherent speed inconveniences of electro-optical systems. Therefore, CMOS-compatible materials with a significant refractive index change under low illumination and a picosecond to femtosecond response time must be available to implement various on-chip nonlinear applications^1^. In this regard, the vital segment of the next-generation integrated optical systems is all-optical devices. Silicon-based optical modulators face fundamental restrictions on the intrinsic silicon properties^2^.

In recent years, epsilon-near-zero (ENZ) materials —i.e., materials for which the real part of the permittivity passes through zero at a specific wavelength—have been introduced as one of the most promising candidates for all-optical elements^1^. The operating range of natural homogeneous ENZ materials, comprising metals^3^ or semiconductors^4^, can be tuned via metasurfaces^5^. These materials present mysterious characteristics such as photon tunneling^6^, highly directional radiation^7^, and perfect absorption^8^.

The main mechanisms for ENZ realization comprise different methods^9–14^ that for semiconductors is through utilizing the free carriers' collective motion at plasma frequency^10^. This mechanism is more commonly employed. By altering free carrier concentrations in heavily doped Si^10^, III–V group semiconductors^15^, metals^9^, and transparent conducting oxides (TCOs)^16^, one can straightforwardly realize ENZ.

The broad transparency spectra of most ENZ materials, from ultraviolet (UV) to far-infrared (FIR), have been experimentally proven, which makes them promising for applications like visible light communications (VLC) and IR telecommunication windows of 1.3, 1.55, and 2 μm^17–20^.

Besides the high electrical conductivity of TCOs, their good optical transparency provides significant possible optical applications such as liquid crystal displays (LCDs) and transparent electrodes on solar cells^21^. The capability of TCOs being highly doped provides the tunability of their intrinsic optical properties^22,23^. Meaningful changes in TCOs' optical properties occur by imposing small electrical or optical excitation. The TCO materials pose transparent dielectric behavior before ENZ occurs and metal-like features after. Not all semiconductors can reach the ENZ in the near-IR or mid-IR wavelengths due to their very high optical permittivities and the limitation in the level of dopant that they can take^24^.

Doped zinc or indium oxides pose low losses due to the small imaginary part of their permittivities^25^ besides offering an ultrafast response time on the scale of femtoseconds at the ENZ because of the corresponding wavelength range of 1.3–1.5 µm^26,27^. In this regard, these doped-oxides, due to their highly nonlinear optical response^4,28,29^, are inexpensive candidates for ENZ applications in the near-infrared region, such as electro- and all-optical switching^26,30^.

Generally, zinc oxide (ZnO) is an attractive TCO due to its wide bandgap of about 3.5 eV, efficiently high conductivity, easy doping, thermal stability in the presence of III group elements, abundance in nature, and nontoxic nature^31,32^. The conductivity of this material can significantly increase by high levels of doping^33^.

One may deposit Al- and Ga-doped ZnO using methods like sputtering^34^, MBE^35^, PLD^26^, CVD^36^, and ALD^37^. Thin films of aluminum-doped zinc oxide (AZO) exhibit electrical and optical properties that vary with deposition conditions (doping level, temperature, and oxygen adsorption during deposition) and post-deposition treatment conditions (oxygen desorption during thermal annealing)^31^.

Due to AZO's non-stoichiometric nature, the deposition conditions largely influence its physical properties. We can tune the ENZ wavelength by controlling the deposition conditions (like changing the Al content, altering the deposition temperature, or varying the film thickness) or the post-deposition thermal annealing conditions^38^. Moreover, adding ZnO buffer layers and protecting layers (e.g., HfO_2_ or Al_2_O_3_) also prevents Zn evaporation upon high-temperature treatment^39^.

Low-loss AZO layers with very high carrier concentration are required to obtain ENZ properties associated with the telecommunication wavelength range (1260–1625 nm) or to secure the transparent biological spectrum (600–1350 nm)^40^. The ALD-grown AZO often holds poor optical properties and deficient carrier concentrations due to low efficiencies in heavily doped cases (> 10^20^ cm^−3^)^39^. For other techniques such as MBE, PLD, CVD, and sputtering, synchronous application of Zn and Al species with accurately controlled flux ratios for a broad range gives rise to uniformly distributed Al or Ga and efficiently highly doped species^41^.

In optoelectronic modulators or switches, by exerting voltage and electrical field on the TCO, a few nanometers accumulation layer appears temporarily at the interface of TCO and dielectric materials^42^. In this mechanism, the energy consumption will be about a few fJ/bits, but RC delay is the limiting factor for modulation speed. Energy consumption and modulation speed are both the primary figures of merits^43^. However, all-optical modulators are excellent options for reaching ultrafast modulation^30^. There is always a trade-off between the modulation speed, insertion loss (IL), and modulation depth (MD)^44,45^. Therefore, scholars continuously try to design the structure with maximum MD and modulation speed. Although the all-optical modulators have absorption/recombination time limitations that influence modulation speed, by low-temperature fabrication techniques, one can control the bulk and surface recombination centers and nanoparticle trapping to reduce the recombination time^46^.

Depositing AZO with extreme oxygen deficiency leaves a large density of oxygen vacancies in the film, providing additional intrinsic carriers and generating deep-level defects^42^. These deep-level defects reduce the carriers' recombination time drastically.

In this work, we propose an all-optical modulator, realizing a highly efficient MD, very low IL, and great modulation speed. Here, we demonstrate the application of an AZO thin film as an optically tunable layer for the modulation of C-band telecommunication window wavelength. Sandwiching AZO film between a distributed Bragg reflector (DBR) and a top patterned metasurface provides the desirable optical properties for the proposed modulator. The fascinating modulation capability of AZO along with its piezoelectric property^47^ can make it an appropriate candidate for applications like birefringence control^48^ and optical polarizer^49,50^.

The rest of the paper is as follows: first, we elaborate on the proposed structure of the modulator, containing its geometry and functionality. Then, we study the optical tunability of the AZO and its dependent properties on the illuminated light, followed by a brief suggested experimental setup to consider its feasibility besides simulation results. The next step includes the results and verification of the ability of the all-optical AZO-based modulator, topped with a metasurface, to earn a promising figure of merit (FOM). The results show superb potential for the designed AZO-based modulator for integration into next-generation all-optical telecommunication systems.

### The proposed structure

The light employed as the controlling signal in the proposed all-optical modulator has photons of energy higher than the bandgap of the active material to excite the valance band electrons to the conduction band through interband excitation. These excess carrier concentrations penetrate the bulk to a certain depth and alter the optical permittivity and refractive index accordingly, shifting the reflectance and absorbance spectra.

**Figure 1:**
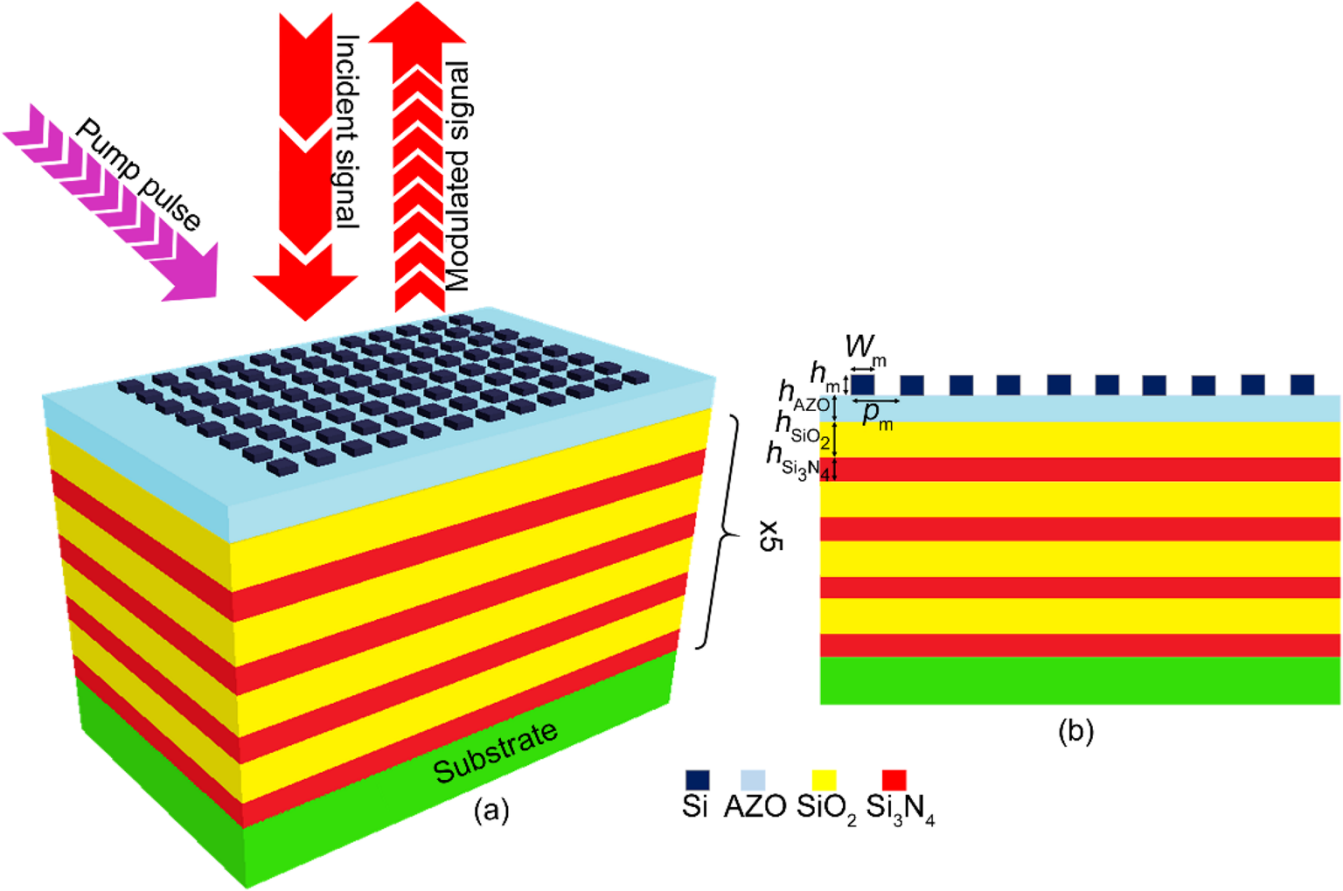
Schematic (**a**) perspective and (**b**) side view of the proposed modulator*.*

Figure 1 illustrates a three-dimensional perspective schematic of the proposed all-optical reflective modulator structure. As depicted in this figure, the proposed modulator consists of a distributed Bragg reflector (DBR) formed by a twenty-pair stack of quarter-wavelength SiO_2_/Si_3_N_4_ deposited on a sapphire substrate. We designed the DBR to reflect the range of incident light almost entirely, assumed as the structure loss. The 120-nm thick oxygen-deprived AZO layer deposited on top of the DBR has an essential role in the tunability of the optical modulator. The metasurface consists of a 2D array of cubic Si gratings patterned on top of the structure, coupling the incident light as a guided-mode resonance (GMR). We have optimized its geometry that highly affects the GMR wavelength according to the procedure we have already described elsewhere^43^ to achieve the critical coupling condition for maximum absorption and minimum reflection at λ = 1310 nm in the modulator OFF state. In other words, when the pump pulse is OFF, the reflectivity at 1310 nm drops to near zero, but in the presence of a pump pulse, it becomes more than 0.9. Table 1 tabulates the physical and geometrical parameters of the structure shown in Fig 1.

**Table 1:** The geometrical and physical parameters used in the designed all-optical modulator.

Symbol	Definition	Value	Units
*p*_m_	Metasurface Pitch	910	nm
*W*_m_	Metasurface horizontal dimension	700	nm
*h*_m_	Metasurface height	43	nm
*h*_AZO_	AZO layer thickness	120	nm
*h*_SiO2_	SiO_2_ slab thickness	226	nm
*h*_Si3N4_	Si_3_N_4_ slab thickness	164	nm
N	Number of SiO_2_/Si_3_N_4_ pairs	20	−
*n*_SiO2_	SiO_2_ refractive index	1.45	−
*n*_Si3N4_	Si_3_N_4_ refractive index	2	−
*m*_0_	Electron rest mass	9.1 × 10^–31^	kg
*ε*_OPT_	AZO relative permittivity at optical frequency	2.8	−

To show how one may employ the proposed modulator in practice, we schematically depicted a suggested experimental setup in Supplementary (Fig. S1).

### Modulation principles

The SiO_2_/Si_3_N_4_ DBR is a perfect mirror for 1305 < *λ* < 1315 nm, with negligible reflectance. In this regard, the AZO layer and the grating topped this layer bear the principal modulation role.

AZO has a metal-like behavior in the infrared spectrum and is highly reflective. On the other hand, in the visible spectrum, AZO is highly transparent and has dielectric-like properties. The intersection of these two regimes occurs at the ENZ.

The optical pump with *λ* = 327.5 nm is incident to the structure. At this wavelength, the photons have enough energy to excite the valence band electrons, changing the carrier concentration of the AZO layer and subsequently modifying the permittivity and refractive index of the AZO^31^. One can calculate the plasma frequency *ω*_p_ via^9^,

1 $$\omega_{p}^{2} = \frac{{n_{{{\text{opt}}}} e^{2} }}{{\varepsilon_{0} \varepsilon_{{{\text{opt}}}} m_{{{\text{eff}}}} }}.$$

Here, ε_opt_ is the optical permittivity, *n*_opt_ is the optically generated carrier density, and *m*_eff_ is the electros’ effective mass. Equation (1) modifies the complex permittivity of the AZO through the Drude-Lorentz model at a near-IR frequency ω^9,51,52^,

2 $$\varepsilon (\omega ) = \varepsilon_{{{\text{opt}}}} (\omega ) + \frac{{\omega_{p}^{2} }}{{ - \omega \left( {\omega - j\,\omega_{\tau } } \right)}} + \frac{{\omega_{L}^{2} }}{{\left( {\omega_{L}^{2} - \omega^{2} } \right) - j\omega \gamma_{L} }},$$

wherein *γ*_L_ and *ω*_τ_ are the damping coefficient and frequency, and *ω*_L_ represents the Lorentz resonance frequency.

**Figure 2:**
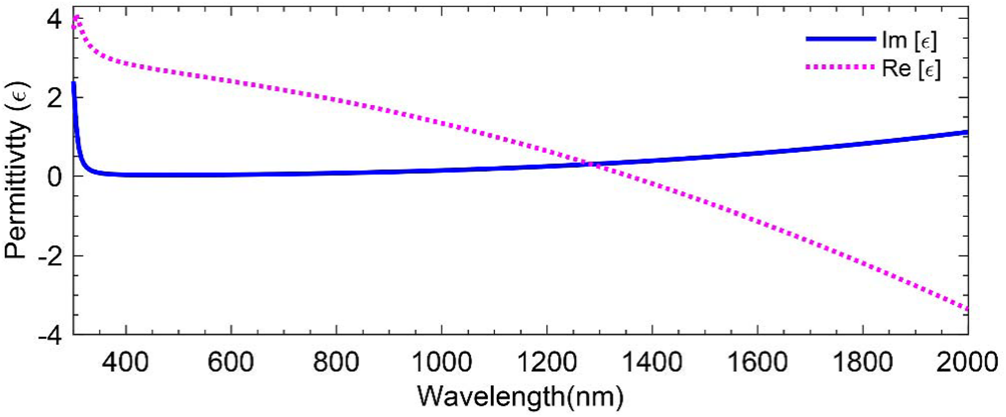
Real and Imaginary part of the permittivity of the AZO through the Drude-Lorentz model.

Figure 2 depicts the real and imaginary parts of the modified permittivity, *ε*(*ω*), calculated for the wavelength range of 300 nm < *λ* < 2000 nm, employing *γ*_L_ = 3.0 × 10^2^ THz, *ω*_L_ = 5.0 × 10^3^ THz, and *ω*_τ_ = 2.0 × 10^2^ THz^9,51,52^ and the fit parameter *ω*_p_ = 2.1 × 10^3^ THz for as-deposited AZO film with Aluminum content of 1.9%^9,51,52^. This figure shows that the ENZ region for the proposed AZO occurs at about *λ* = 1300 nm, making it a promising candidate for communication window C.

The wavelength-dependent modulation depth of the modulator can be determined from its reflection spectra in the OFF (*R*_OFF_ (λ)) and ON (*R*_ON_ (λ)) states obtained via numerical simulation by COMSOL Multiphysics^53^

3 $${\text{MD}} (\lambda ) = 10\log \frac{{\max \left[ {R_{{{\text{ON}}}} (\lambda ){\text{ and }}R_{{{\text{OFF}}}} (\lambda )} \right]}}{{\min \left[ {R_{ON} (\lambda ){\text{ and }}R_{{{\text{OFF}}}} (\lambda )} \right]}}.$$

Moreover, the modulator insertion loss,

4 $${\text{IL}} (\lambda ) = 10\log \left\{ {\max \left[ {R_{{{\text{ON}}}} (\lambda ){\text{ and }}R_{{{\text{OFF}}}} (\lambda )} \right]} \right\},$$

is also of practical importance because it directly impacts the system's efficiency^54^.

As mentioned before, it is difficult to further improve the modulation speed in electro-optical modulators due to the functional response time limitations of the electrical section^55–57^. Nevertheless, all-optical modulation can overcome the modulation rate limitations by using one light beam to control the transmission/reflection of another light beam^30,58^. Several papers have reported all-optical modulation schemes with high speeds of 200 GHz based on graphene devices, in which a graphene sheet covers each structure. As a result of the required low insertion loss, these schemes have relatively low modulation depth or modulation efficiency^59–61^.

One of the essential characteristics of TCOs is the maximum achievable change in the optical properties, which in this case, is the result of excess carrier generation.

By gating the active layer in electro-optical modulation, only a few nanometers thick accumulation layer forms at the TCO/dielectric interface. Nonetheless, in all-optical modulation, the optical excitation is more than ten times throughout the bulk^26^.

## Results and discussion

We employed finite element methods (using COMSOL Multiphysics) to simulate the optical behavior of the proposed AZO layer under pump pulse and also analyze the entire device under optically ON and OFF conditions, along with the application of MATLAB for MD and IL calculations.

**Figure 3:**
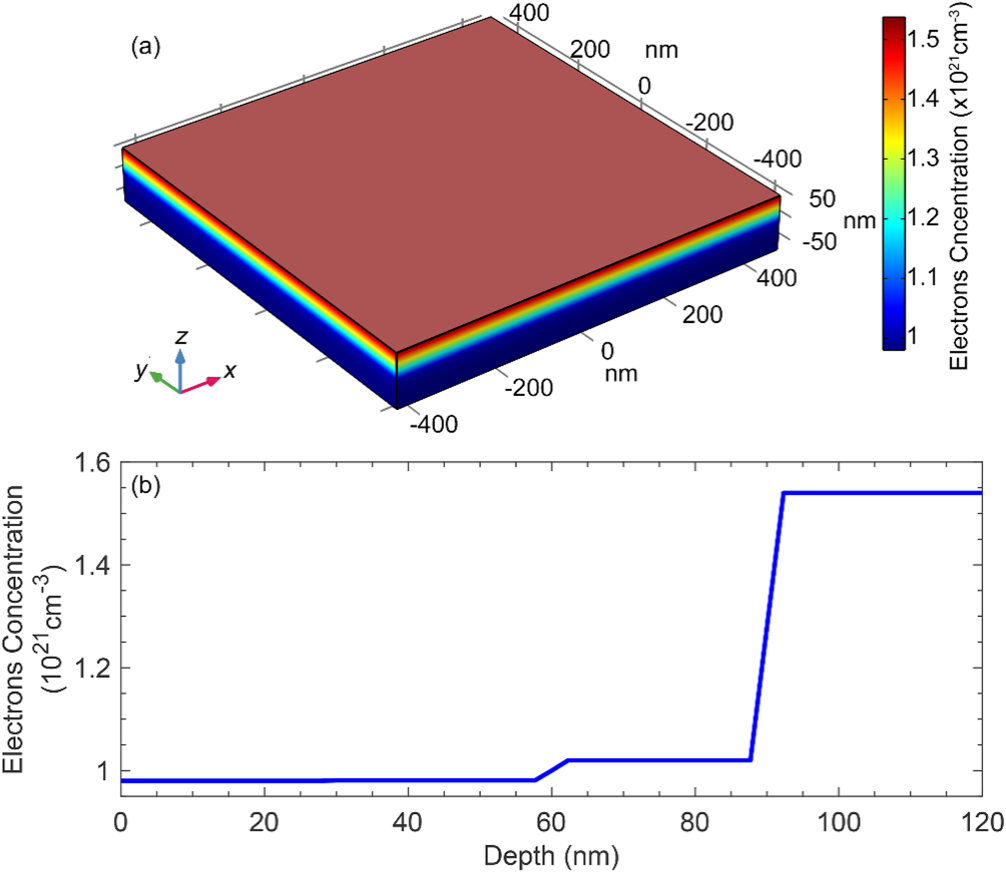
(**a**) 3D profile of the carrier concentration within a 900 × 900 × 120 nm AZO layer optically pumped at *λ* = 327.5 nm with the intensity of 2.41 mJ∙cm^−2^, (**b**) The maximum carrier concentration within the depth of the AZO layer (i.e., along the *z*-axis).

In this virtual experiment, we illuminate the metasurface simultaneously by the light signal of wavelength *λ*_s_ = 1310 nm and a quarter wavelength optical pump pulse (λ_p_ = 327.5 nm). The signal's photons energy is less than the AZO layer bandgap. Hence, in agreement with the experimental studies^18–20^, reporting AZO with ~ 80% transparency for a bandwidth including 1310 nm, it enjoys the same transparency for the incident signal photons. Notice that the fit parameters used for as-deposited AZO film with Aluminum content of 1.9%^9,51,52^ took the actual AZO layer transparency into account. On the other hand, the AZO layer bandgap is smaller than the pump photons' energy, absorbing them. This absorption can result in the generation of excess carriers of ~ 4×10^20^ cm^−3^, which is essential for the modulation operation, using the total pulse fluence of only a few milli-Joules (2.41 mJ∙cm^−2^). Figure 3a depicts a 3D profile of the excess carrier concentration generated within the volume of an AZO unit cell of dimensions 900 × 900 × 120 nm, optically pumped at λ = 327.5 nm with the fluence of 2.41 mJ∙cm^−2^. Figure 3b shows the penetration depth of the maximum carrier concentration carriers generated within the 120-nm thick AZO layer. Our calculations show the estimated average carrier concentration within the top 60 nm of the AZO layer under optical pump pulse irradiation is ~ 1.38×10^21^ cm^−3^ (with a maximum of ~ 1.54×10^21^ cm^−3^). The carrier concentration within the bottom 60 nm almost equals the initial carrier concentration. The difference in the carrier concentrations changes the plasma frequency of the top segment of the AZO layer, Eq. (1), hence changing its refractive index (Eq. 2).

**Figure 4:**
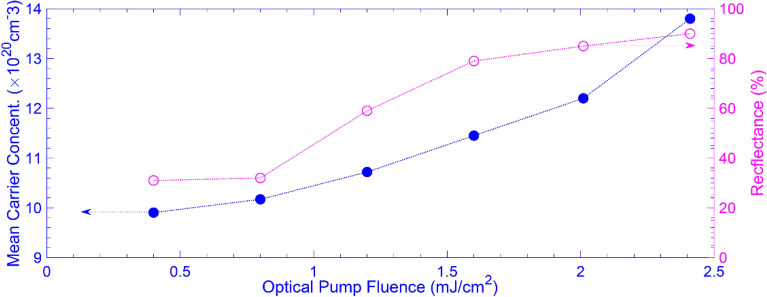
The mean carrier concentration in the AZO layer (solid blue circles read from the left axis) and the modulator reflectance spectra (open pink circles read from the right axis) versus the optical pump fluence.

Here, we demonstrate how the pump fluence affects the mean carrier concentration of the AZO layer, thereby altering the AZO layer's permittivity through Eqs. (1) and (2). The solid blue circles in Fig. 4 (the left axis) depict the resulting mean carrier concentration versus the pump fluence. On this curve, we can see the mean carrier concentration increases in a nonlinear manner as the pump fluence increases, shifting the GMR of the device to its ON state. As a result, the reflected signal varies at each optical pump fluence, as shown by the open pink circles (the right axis).

**Figure 5:**
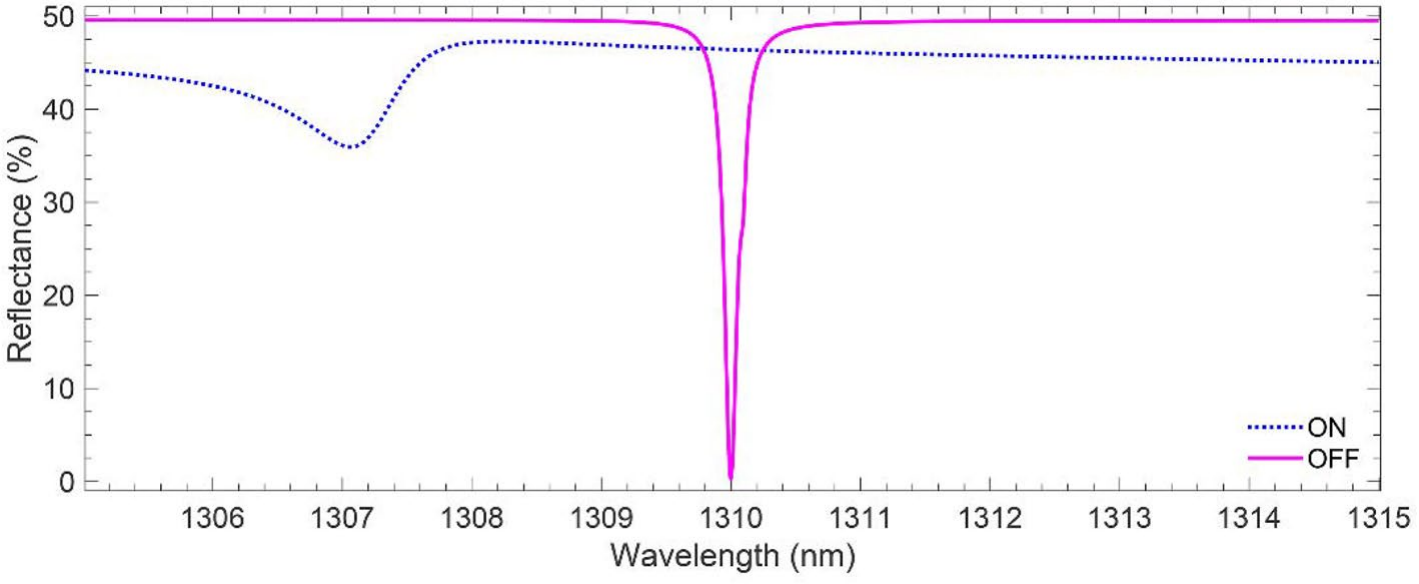
Reflectance spectra of the modulator for ON and OFF states at 1305 < *λ* < 1315.

Figure 5 shows the numerical results for the spectral response of the proposed modulator in the OFF and ON states, obtained via the COMSOL Multiphysics simulations. The solid magenta line demonstrates that when the pump pulse is OFF, the modulator structure reflects the incident signal in a vast part of the wavelength range of 1305 < λ < 1315), except in a narrow bandwidth (FWHM = 0.13 nm) about *λ* = 1310 nm, where *R*_OFF, min_ *≈* 0.8%. It is worth mentioning that this nearly perfect (99.2%) absorption is due to the appropriately engineered Si metasurfaces satisfying the critical coupling condition at λ = 1310 nm. Moreover, any deviation in the metasurface geometry from the dimensions given in Table 1, alters the narrow linewidth at 1310 nm^43^. The linewidth of the reflectance in the OFF state indicates the device quality factor *Q* > 10,000. Such a narrow bandwidth profile with a sharp peak agrees with the result of an experimental study, showing the feasibility of obtaining narrow linewidth and high-peak response for the guided mode resonance reflection filters^62^. For demonstrating the effects of DBR and the AZO layer on the modulator’s total reflectance, we assessed the optical response—i.e., reflectance, transmittance, and absorbance—of the DBR and the DBR topped with the AZO layer as plotted in Fig. S2 of Supplementary. Turning on the pump pulse with photons energy higher than the AZO layer bandgap generates excess carriers, modifying the AZO layer plasma frequency and permittivity. According to the data shown in Fig. 4b and Eq. (1), the excess carrier concentration generated within the top 60 nm of the AZO layer (ON state) changes the permittivity of this segment from *ε*_OFF_ ≈ 0.831+0.025*i* to *ε*_ON_ ≈ − 0.213 + 0.055*i*. The resulting decrease in the Re [*ε*(*ω*)] causes a blue shift in the resonant frequency of the designed Si metasurfaces. On the other hand, the increase in the Im [ε(ω)] makes the AZO layer more absorptive. These can be observed from the blue dots in Fig. 5, indicating a 3-nm blue shift in the reflection's minimum of the ON state (*R*_ON, min_≈ 0.719). The solid magenta line in Fig. 5 represents the spectra for the OFF (*R*_OFF_) state.

**Figure 6:**
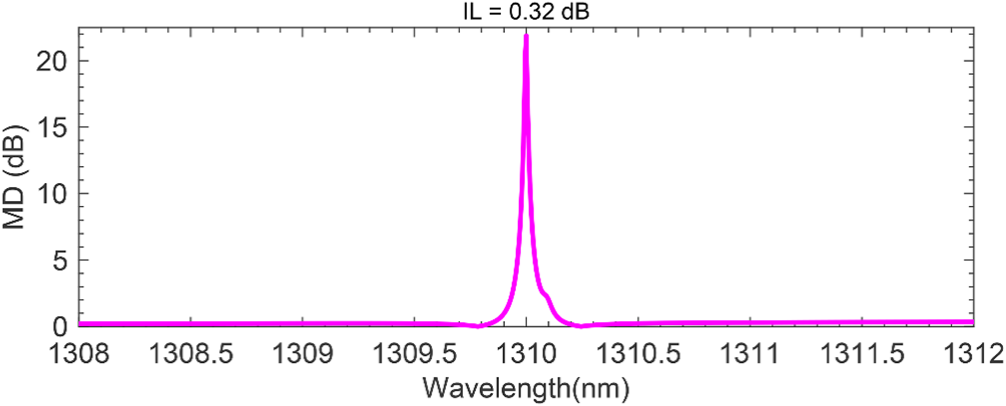
MD (*λ*) and IL at *λ* = 1310 nm.

Employing Eq. (3) and using the data shown in Fig. 5, we have calculated the modulator MD spectrum (Fig. 6). The results show the proposed modulator enjoys the modulation depth of MD ≈ 22 dB at the resonant wavelength of *λ* = 1310 nm, with an acceptable insertion loss as low as IL = 0.32. In most cases, like in high-speed interconnections or high-sensitivity sensors, a high modulation depth of > 7 dB is desirable. Hence, the simultaneous high MD and low IL values demonstrated by the proposed all-optical AZO-based modulator topped with Si metasurfaces make it unique among other all-optical counterparts. According to the composition of the AZO layer specified in this study, a modulation speed greater than 1 THz with a relaxation time of fewer than 500 fs is achievable^26^.

## Conclusion

Conquering the RC delay of electro-optic modulators should be mentioned as one of the main priorities all-optical modulators possess. In this numerical study, by recruiting a 120 nm AZO layer over a 20-pair Si_3_N_4_/SiO_2_ DBR, appropriately designed for near-perfect reflection within a vast part of the wavelength range including 1305 nm < *λ* < 1315 nm except for a narrow band around *λ* = 1310 nm, that is covered with 700 × 700 × 43 nm Si metasurfaces, we obtained a highly efficient modulator. An optical pump pulse at *λ* = 327.5 nm is irradiated to the AZO layer to change the intrinsic carrier concentration of the top 60 nm of this layer from 9.8 × 10^20^ to 1.38 ×10^21^ on average. Carrier concentration variation leads to tuning the resonant wavelength of the Si metasurfaces and consequently shifts the reflection spectrum of the modulator. The simulation results show the proposed all-optical modulator enjoys modulation depth and insertion loss of MD = 22 dB and IL = 0.32 dB, with a pump fluence level of 2.41 mJ∙cm^−2^.

## Supplementary Information

Supplementary Information.

## Data Availability

Data underlying the results presented in this paper are not publicly available at this time but may be obtained from the corresponding author upon reasonable request.
