# Supplementary material for: All-optical AZO-based modulator topped with Si metasurfaces
Source: Sci Rep. 2022 Dec 13;12:21490. doi: 10.1038/s41598-022-25991-9 (PMC9748133; doi:10.1038/s41598-022-25991-9)
Supplement: Supplementary file 1 — Supplementary Information. [file 41598_2022_25991_MOESM1_ESM.docx]

**Supplementary**

**All-Optical AZO-based Modulator Topped with Si Metasurfaces**

**Sareh Vatani, Behdad Barahimi, and Mohammad Kazem Moravvej-Farshi^*^**

Nano Plasmo-Photonic Research Group, Faculty of Electrical and Computer Engineering, Tarbiat Modares University, Tehran 1411713116, Iran

# * Email: [moravvej@modares.ac.ir](mailto:moravvej@modares.ac.ir)

# Suggested optical setup

Figure S1 shows a practicable setup that makes us better understand and evaluate the feasibility of the proposed structure (Figure 1). The first beam splitter (BS1) splits the intensity of a 1310-nm laser light source into two equal branches, one of which passes through two BBO crystals (BBO1 and BBO2), generating the second and third harmonics, in turn, yielding 655 nm and 327.5 nm wavelengths, respectively. The role of filters F1 and F2 after BBO1 and BBO2 crystals are to filter any undesired wavelength at their corresponding stages. The resulting third harmonic (327.5 nm) acts as the pump light finally focused onto the designed modulator. The second split light branch is directed to a one-way filter (F3) and then focused onto the modulator as the probe. The role of F3 is to prevent the interference of any back-reflected light with the incoming beam through the second split branch. The detector D collects the reflected 1310 nm light via the second beam splitter BS2.


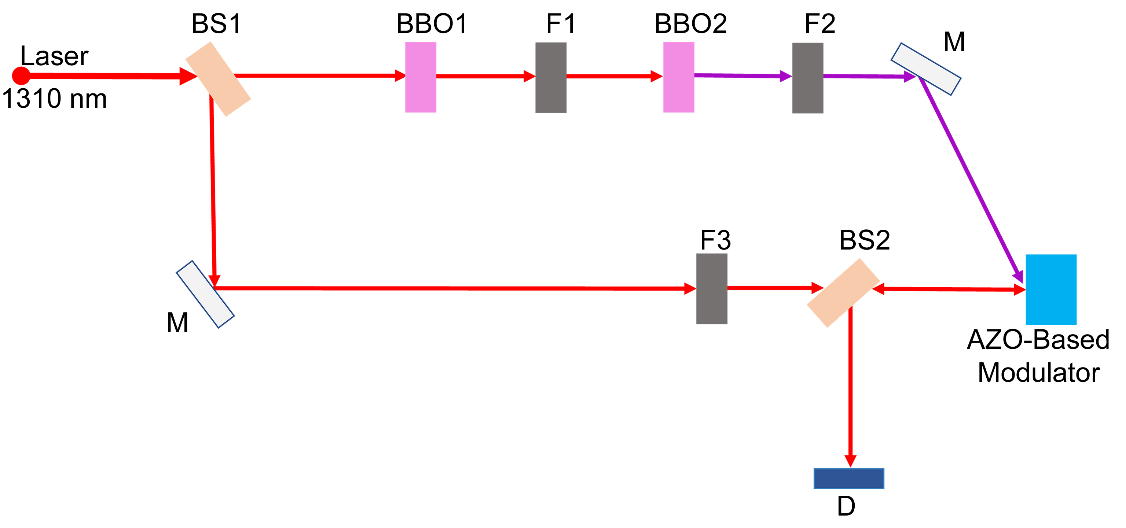


**Figure S1**. Suggested experimental setup: BS: beam splitter, M: mirror, BBO: beta barium borate crystal, F: filter, D: detector.

# Optical response

The modulator's optical response is shown in Fig. 5 of the manuscript. Nevertheless, for a better demonstration of the effect of the segments of the modulator, we show the optical response — i.e., reflectance, transmittance, and absorbance — of the DBR alone (Fig. S2(a)) and the DBR topped with the AZO layer (Fig. S2(b)), here.


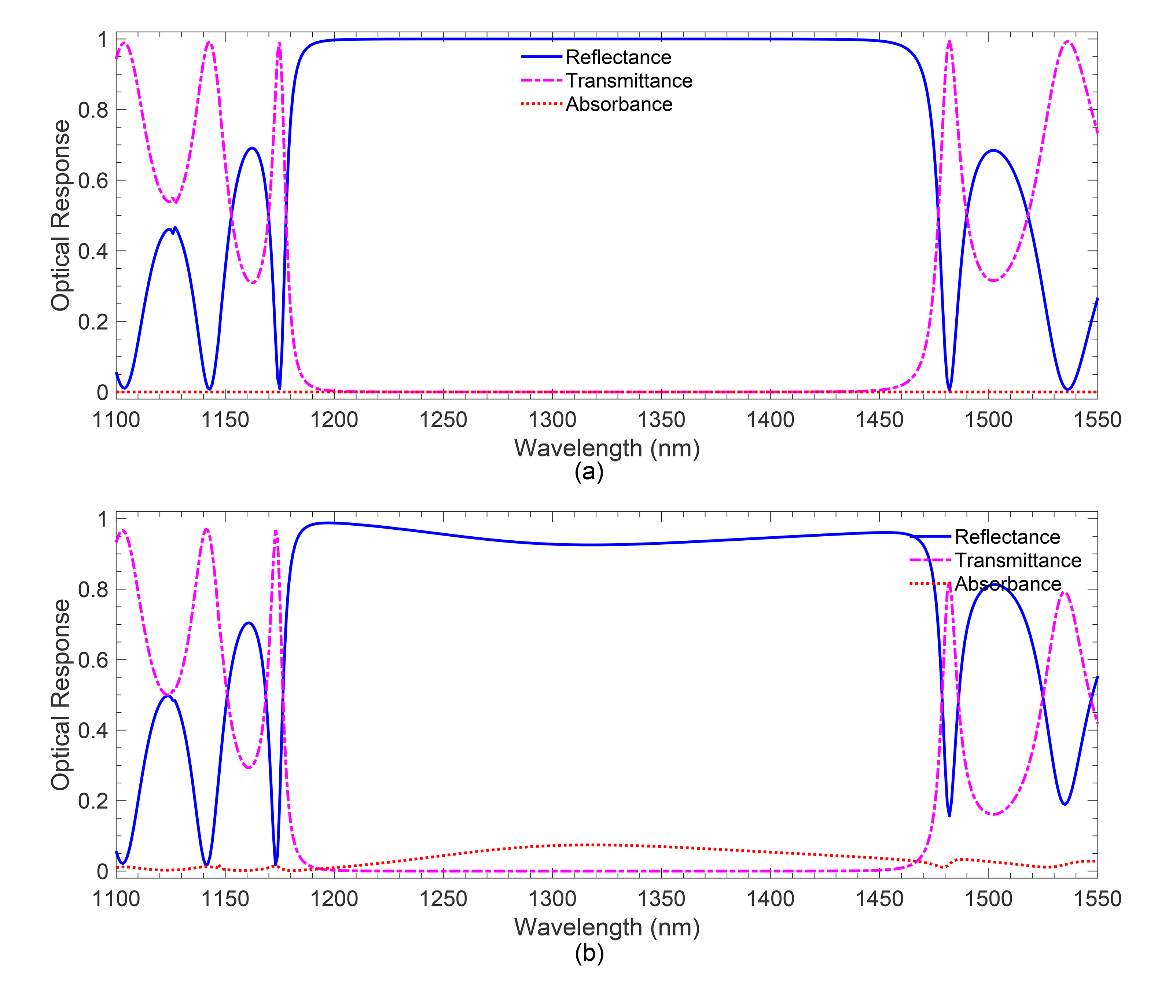


**Figure S2**. Optical response of the (a) DBR and (b) DBR topped with the AZO layer.
